# Piezo ion channels in the digestive system: Mechanotransduction pathways and therapeutic targeting strategies

**DOI:** 10.1016/j.gendis.2025.101925

**Published:** 2025-11-07

**Authors:** Xiangyun Yan, Weijian Zeng, Peitao Ma, Junpeng Yao, Tingting Ma, Ying Li

**Affiliations:** a School of Acupuncture and Tuina, Chengdu University of Traditional Chinese Medicine, Chengdu, Sichuan 611137, China; b Key Laboratory of Acupuncture for Senile Disease, Chengdu University of Traditional Chinese Medicine, Ministry of Education, Chengdu, Sichuan 611137, China; c Center of Preventive Medicine, Hospital of Chengdu University of Traditional Chinese Medicine, Chengdu, Sichuan 610075, China

**Keywords:** Digestive system, Pathology, Physiology, Piezo1, Piezo2

## Abstract

Piezo ion channels, notably Piezo1 and Piezo2, are key mechanosensors that transduce mechanical forces into intracellular signals, playing indispensable roles in digestive physiology. These channels regulate essential functions such as intestinal motility, epithelial barrier integrity, bile secretion, and host–microbiota balance. Emerging evidence links aberrant Piezo signaling to a wide range of gastrointestinal disorders, including functional bowel diseases, inflammatory conditions, and digestive cancers. However, translating these insights into therapeutic applications remains challenging. Most current findings are derived from animal models or *in vitro* studies, which do not fully recapitulate human tissue complexity. Advanced human-relevant platforms, such as organoids and organ-on-a-chip systems, are needed to bridge this translational gap. Furthermore, Piezo1 and Piezo2 play both overlapping and distinct roles in gastrointestinal pathophysiology, necessitating selective modulation strategies. While Piezo1 promotes processes such as epithelial remodeling and tumor invasion via pathways like RhoA/ROCK and YAP/TAZ, Piezo2 is more associated with sensory neuron activity, immune modulation, and tumor aggressiveness. The lack of specific agonists and inhibitors, especially for Piezo2, further limits its clinical translation. Lastly, Piezo channels are deeply integrated into complex molecular networks involving focal adhesions, cytoskeletal dynamics, and transcriptional regulation. This review synthesizes current advances in the mechanobiology of Piezo channels within the digestive system and highlights future directions for mechanistically-informed, Piezo-targeted therapies.

## Introduction

Mechanotransduction is a fundamental biological process through which cells convert mechanical stimuli into electrochemical signals, thereby enabling dynamic cellular responses to environmental changes.^1^ This process plays a critical role not only in sensory perception, such as touch and hearing, but also in essential physiological functions, including vascular tone and blood pressure regulation.^2^ In 2010, Coste et al identified the Piezo family of mechanosensitive ion channels, encoded by the Piezo1 (Fam38A) and Piezo2 (Fam38B) genes, marking a key breakthrough in the field.^3^ Their discovery has significantly advanced our understanding of how mechanical forces are transduced at the cellular level, with broad implications for health and disease.

Piezo1 and Piezo2 exhibit unique trimeric, propeller-like structures that directly facilitate ion flux in response to mechanical stimuli.^4^^,^^5^ These channels display rapid activation and inactivation dynamics, enabling cells to finely tune the intracellular signaling cascades involved in embryonic development, cell proliferation, and differentiation.^6^, ^7^, ^8^ Dysregulation of Piezo channel activity has been implicated in a spectrum of pathological conditions, including hypertension, chronic pain, and tissue fibrosis.^9^, ^10^, ^11^ These findings highlight their emerging potential as mechanosensitive therapeutic targets in diseases associated with aberrant mechanical environments.

Within the digestive system, Piezo channels serve as crucial mechanotransducers that convert physical forces, such as luminal flow and tissue distension, into biochemical signals.^12^ Through this process, they regulate gastrointestinal motility, epithelial barrier integrity, and visceral sensitivity.^13^, ^14^, ^15^ Aberrant Piezo signaling has been associated with various gastrointestinal disorders, including inflammatory bowel disease, pancreatitis, and gastrointestinal malignancies, underscoring its importance in both gut physiology and pathophysiology.^16^, ^17^, ^18^

In this review, we provide a comprehensive overview of recent advances in the structural biology, mechanotransduction mechanisms, and physiological functions of Piezo channels within the digestive tract. We further discuss their involvement in the pathogenesis of gastrointestinal diseases and examine their potential as novel targets for mechanosensation-based therapeutic strategies.

## Mechanisms of activation and inactivation of Piezo ion channels

**Figure 1:**
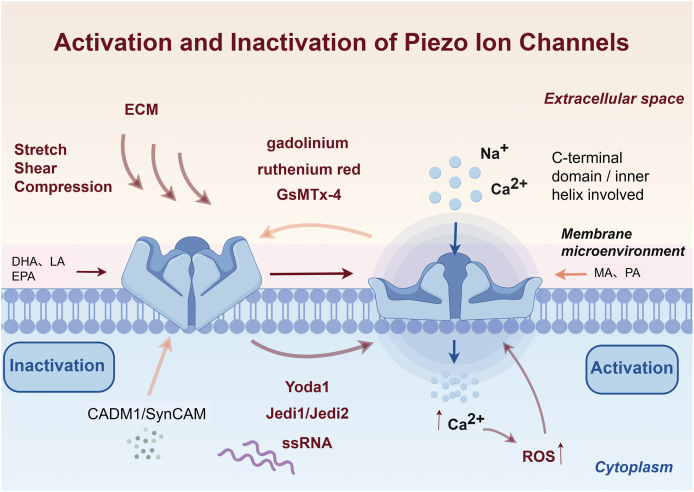
Piezo channels are activated by mechanical cues such as stretch, shear stress, and compression, often transmitted through extracellular matrix (ECM) components, which induce conformational changes in the C-terminal domain and inner helix to open the pore and permit Na^+^ and Ca^2+^ influx. Excessive Ca^2+^ entry can lead to calcium overload and ROS generation, linking sustained mechanical stress to oxidative modulation of channel gating. Channel activity is further shaped by the membrane microenvironment and protein interactors: fatty acids such as DHA, LA, and EPA enhance Piezo activity, while margaric acid (MA) and phosphatidic acid (PA) inhibit it, and CADM1/SynCAM modulates inactivation kinetics. Pharmacological modulators include non-selective blockers (gadolinium, ruthenium red, GsMTx-4) and Piezo1-selective agonists (Yoda1, Jedi1, Jedi2), while fecal single-stranded RNA (ssRNA) has been proposed as a natural ligand although remains controversial. To avoid overstimulation, Piezo channels normally transition to an inactivated state, thereby supporting sustained mechanotransduction in sensory neurons.

Piezo ion channels are essential mediators of mechanosensation, characterized by rapid transitions between inactive and active states in response to mechanical stimuli.^19^ Activation of these channels occurs through mechanical forces transmitted by the cytoskeleton, facilitating the influx of calcium (Ca^2+^) and sodium (Na^+^) ions, which induce conformational changes that open the pore^20^ (Fig. 1). However, when Ca^2+^ entry becomes excessive, calcium overload occurs, which disrupts the mitochondrial membrane potential and promotes reactive oxygen species (ROS) generation. This Ca^2+^–redox coupling was directly demonstrated by optical tweezer experiments in single endothelial cells, where picoNewton-scale forces activated Piezo1 (with TRPV4 participation) to drive Ca^2+^ influx and subsequent NO/ROS production.^21^ ROS further destabilize channel gating by oxidizing cysteine and methionine residues within intracellular domains, with methionine oxidation playing a predominant role, as recently demonstrated in Piezo1 under oxidative conditions.^22^ These oxidative modifications, together with lipid peroxidation, prolong channel activity and impair inactivation. This Ca^2+^ overload–ROS feedback loop establishes a direct molecular link between sustained mechanical stress and pathological outcomes such as chronic inflammation, fibrosis, and cell death.

Piezo1 exhibits high sensitivity to various mechanical stresses, such as stretching, compression, and shear stress, and its sensitivity is modulated by extracellular matrix components, including collagen IV.^23^, ^24^, ^25^ Increased matrix stiffness further enhances Piezo1 activation, consistent with the integrin–cytoskeleton mediation of the stiffness effect.^26^ To prevent cellular overstimulation by continuous mechanical inputs, Piezo channels normally inactivate rapidly.^3^ The inactivation process, closely associated with the channels' C-terminal extracellular domain and the inner pore helix, ensures precise control over cellular responses.^27^ Compared with Piezo1, Piezo2 exhibits slower inactivation kinetics, enabling prolonged mechanotransduction in sensory neurons.^3^^,^^28^ Dysfunction in these inactivation mechanisms is linked to persistent mechanosensitivity, contributing to pathological conditions such as chronic pain, fibrosis, and cardiovascular disorders.^29^ Beyond the intrinsic structure, proximal interactors can tune inactivation; for example, CADM1/SynCAM modulates Piezo1 inactivation dynamics.^30^ Piezo2 gating is also subject to voltage control; mutation at residue R2756 relieves voltage block and lowers the mechanical activation threshold, providing a mechanistic basis for hyperalgesia.^31^

The membrane microenvironment further shapes Piezo gating. Specific fatty acids differentially modulate channel behavior: docosahexaenoic acid (DHA) preferentially enhances Piezo1 activity; margaric acid (C17:0) inhibits both Piezo1 and Piezo2; linoleic acid (C18:2) enhances both; and eicosapentaenoic acid (EPA) shortens inactivation time constants in wild-type and gain-of-function variants. In addition, phosphatidic acid (PA), generated by phospholipase D (PLD), acts as an endogenous negative regulator of Piezo2 but not Piezo1, while the transmembrane channel-like protein 7 (TMC7) functions as a suppressor of Piezo2-dependent mechanotransduction in sensory neurons, underscoring isoform-specific crosstalk among mechanotransducers.^32^^,^^33^

Pharmacological tools remain central for dissecting gating. Non-specific blockers of mechanically activated channels, including gadolinium, ruthenium red, and GsMTx-4, inhibit both Piezo1 and Piezo2. Small-molecule screening has yielded Piezo1-selective activators such as Yoda1 and its analogs Jedi1/Jedi2, which lower the activation threshold independently of external force.^34^^,^^35^ By contrast, Piezo2-selective activators remain elusive. More recently, fecal ssRNA has been suggested as a potential natural agonist for Piezo1; however, its reproducibility remains controversial, and the precise mechanism by which it might influence Piezo gating has not been established.^36^^,^^37^ Such discrepancies may arise from differences in experimental systems, the presence of confounding microbial or metabolic components in fecal extracts, or indirect effects mediated by immune signaling rather than direct channel binding. Clarifying whether ssRNA represents a bona fide endogenous Piezo agonist will require rigorous validation across model systems and structural approaches.

These diverse regulatory inputs highlight that Piezo gating is not governed by a single determinant but rather emerges from the integration of mechanical, redox, lipid, and protein cues. Clarifying the relative contribution and interplay of these factors across different cell types remains an unmet challenge.

## Mechanotransduction pathways of Piezo ion channels

**Figure 2:**
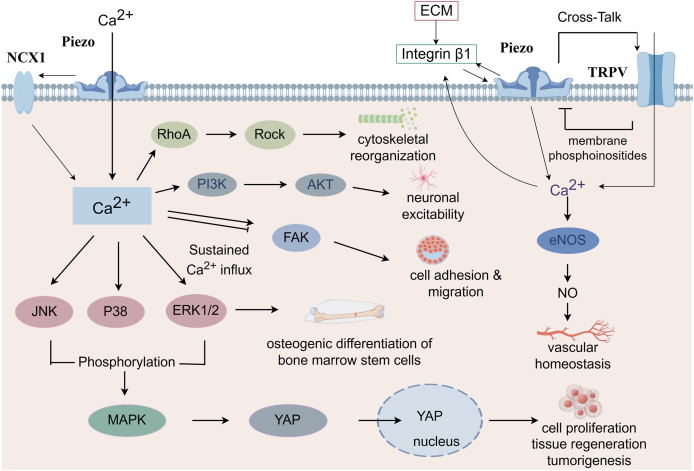
Activation of Piezo ion channels by mechanical stimuli induces intracellular Ca^2+^ influx, which triggers multiple mechanotransduction cascades. Calcium entry activates RhoA/ROCK signaling to drive cytoskeletal reorganization, while sustained Ca^2+^ influx engages focal adhesion kinase (FAK) to promote cell adhesion, migration, and osteogenic differentiation of bone marrow stem cells. Parallel activation of JNK, P38, and ERK1/2 phosphorylates the MAPK pathway and regulates YAP nuclear translocation, thereby linking Piezo activity to cell proliferation, tissue regeneration, and tumorigenesis. Partner interactions further refine these outcomes: integrin β1 couples extracellular matrix mechanics to Piezo1-mediated Ca^2+^ signaling, reinforcing adhesion and migration programs; NCX1, operating in reverse mode, cooperates with Piezo1 to amplify Ca^2+^ overload; and crosstalk with TRPV channels modulates Ca^2+^ flux, where Piezo-dependent phosphoinositide remodeling facilitates TRPV4 activation, stimulating eNOS and nitric oxide (NO) release to support vascular homeostasis.

Upon activation by mechanical stress, Piezo channels undergo conformational rearrangements that permit the entry of Ca^2+^.^20^ This calcium increase not only initiates depolarization and downstream cascades such as MAPK, Hippo-YAP, and RhoA/ROCK signaling but also interacts functionally with other ion channels including TRP and voltage-gated Na^+^/K^+^ channels. Such crosstalk amplifies electrical excitability and diversifies signaling outputs. In doing so, Piezo activity is embedded into broader mechanosensory networks that regulate cytoskeletal remodeling, barrier function, and inflammatory responses (Fig. 2).

### Calcium signaling and pathway integration

Calcium influx mediated by Piezo channels significantly elevates intracellular Ca^2+^ concentrations, activating multiple essential signaling pathways. Notably, JNK/p38/ERK phosphorylation activates the MAPK pathway to regulate YAP nuclear translocation, playing a pivotal role in mediating inflammatory responses and stress adaptation.^38^ Advanced imaging technologies, such as fluorescence resonance energy transfer (FRET)-based biosensors, have elucidated a biphasic activation pattern of focal adhesion kinase (FAK) signaling induced by Piezo1, reflecting dynamic cellular responses to mechanical stimuli.^39^ Furthermore, the C-terminal region of Piezo1, containing an R-Ras binding domain, critically regulates calcium influx and downstream activation of the ERK1/2 pathway, which is vital for the osteogenic differentiation of bone marrow stem cells.^40^

Piezo2 likewise mediates calcium-dependent mechanotransduction across multiple systems. In sensory neurons, Piezo2-triggered Ca^2+^ influx activates the MAPK and PI3K–Akt pathways to regulate excitability and pain sensitivity. Recent studies have extended this paradigm: precision magnetic stimulation of the paraventricular nucleus (PVN) increases Piezo2 expression in oxytocinergic neurons, where Ca^2+^ influx activates oxytocin and PI3K–Akt signaling, contributing to behavioral rescue in autism-like mice.^41^ Beyond the role in neuronal contexts, Piezo2 governs developmental processes. During tooth root morphogenesis, Piezo2^+^ sensory neurons convert mechanical forces into Ca^2+^-driven secretion of platelet-derived growth factor A (PDGFA), which directs mesenchymal cell fate and organogenesis.^42^

Together, these findings establish Ca^2+^ influx as the central initiating event for both Piezo1 and Piezo2 signaling, while their downstream repertoires diverge depending on the cellular context. In structural cells, Ca^2+^ entry primarily engages MAPK-YAP, FAK, and ERK signaling, whereas in neurons, it drives the PI3K-Akt and PDGFA pathways, underscoring the broad mechanotransduction capacity of Piezo channels.

### Modulation of cytoskeletal dynamics

Piezo channels critically regulate cytoskeletal dynamics through canonical Ca^2+^-RhoA/ROCK signaling, which governs actin remodeling and contractility under mechanical stress.^43^ Recent evidence emphasizes reciprocity: actin filaments reorganized by microscale geometrical constraints can in turn alter local membrane tension and tune Piezo1 gating.^44^^,^^45^ At the molecular level, Piezo1 anchoring to the actin cytoskeleton via cadherin–β-catenin provides a direct structural link between adhesion, cytoskeletal tension, and channel gating, supporting a “force-from-filament” model that complements the classical “force-from-lipid” paradigm.^46^ Integrin-based focal adhesions further bridge extracellular stiffness with intracellular cytoskeletal forces, coupling extracellular matrix (ECM)-derived mechanics to Piezo1 gating and reinforcing its role as a central hub in mechanotransduction.^47^

Functionally, Piezo1-mediated cytoskeletal remodeling is highly context dependent. In epithelial cells, Piezo1 activation drives a DUOX2-H_2_O_2_-FER pathway that coordinates lamellipodia dynamics and accelerates wound closure.^48^ In immune cells, shear stress-induced Piezo1 activation engages calpain to reorganize actin, facilitating neutrophil extracellular trap (NET) formation and amplifying inflammation.^49^ In neurons, Piezo1 at growth cones senses substrate stiffness and promotes axon regeneration through a Ca^2+^-CaMKII-FAK-actin cascade.^50^ These divergent outcomes, epithelial repair, immune activation, and neuronal plasticity, illustrate the versatile integration of mechanical cues into cell type-specific programs.

Beyond the intracellular cytoskeleton, the ECM provides the upstream mechanical context that tunes Piezo activity. Variations in substrate stiffness or microscale geometry reshape actin organization and membrane tension, thereby adjusting Piezo1 gating sensitivity.^44^ In developmental systems, Piezo2 also mediates ECM-guided processes: moderate stiffness promotes progenitor expansion via integrin–F-actin–YAP signaling, whereas increased stiffness drives differentiation through Piezo2-dependent Ca^2+^ influx.^51^ These findings position the ECM-cytoskeleton-Piezo axis as a self-reinforcing mechanotransduction loop that bridges extracellular mechanics with intracellular remodeling.

Although less extensively studied, Piezo2 contributes to cytoskeletal regulation in restricted contexts. Its intrinsically disordered intracellular domains fine-tune force sensitivity; the IDR5 linker transmits cytoskeleton-applied forces without affecting stretch sensitivity.^52^ In dorsal root ganglion neurons, Piezo2 physically interacts with β-actin and TMC7, indicating that its gating requires cytoskeletal coupling.^33^ Thus, Piezo1 broadly organizes cytoskeletal remodeling across systems, while Piezo2 serves as a context-specific tuner, together exemplifying Piezo channels as both sensors of mechanical stress and regulators of cytoskeletal architecture.

### Cross-talk with signaling modules

Piezo channels operate within broader signaling modules, where their Ca^2+^ influx is shaped, amplified, or buffered by partner molecules. This modular integration determines whether mechanical stress stabilizes homeostasis or drives pathology.

In vascular and epithelial contexts, Piezo1 interacts bidirectionally with TRP channels but has contrasting outcomes. Piezo1-TRPV4 coupling via PLA_2_ signaling amplifies Ca^2+^ influx and activates eNOS, promoting vasodilation and vascular protection.^53^^,^^54^ Conversely, the Piezo-TRPV1 interaction suppresses Piezo currents through Ca^2+^-dependent phosphoinositide depletion, attenuating nociceptive mechanotransduction.^55^^,^^56^ These opposing results illustrate that Piezo-TRP crosstalk is context-dependent rather than universally synergistic, resolving the apparent paradox that the same partners can either magnify or restrain mechanosensitivity.

Beyond TRPs, Piezo1 couples with transport systems, again resulting in dual outcomes. In bladder interstitial Cajal-like cells, Piezo1 interacts with NCX1 in its reverse mode, amplifying Ca^2+^ overload and driving hyperactivity in chronic cystitis.^57^ In contrast, in red blood cells, Piezo1 integrates with PMCA and the Gardos K^+^ channel to form a “transporter trio”, where transient Ca^2+^ influx during capillary transit is balanced by PMCA extrusion and Gardos-mediated K^+^ efflux, ensuring volume stability.^58^ This comparison underscores how the same ion channel can destabilize or safeguard physiology depending on its signaling partners.

Piezo1 also engages receptor–channel modules that link mechanical sensing to immune and adhesion pathways. In enterochromaffin cells, the association with TLR4 enables LPS-induced Piezo1 activation, enhancing 5-HT release and contributing to intestinal motility dysfunction.^59^ In bladder carcinoma, Piezo1 and integrin β1 form a positive feedback loop: Piezo1-mediated Ca^2+^ influx drives integrin recycling and macropinocytosis, while stabilized integrin β1 reinforces Piezo1 activity, collectively boosting tumor peptide uptake.^60^ By contrast, in hair follicle stem cells, Piezo1 couples with E-cadherin, where piconewton-scale pulling elicits localized Ca^2+^ flickers that activate AP1/NFATC1 transcriptional programs to maintain stem cell quiescence.^61^ These examples show how Piezo–adhesion crosstalk can be hijacked for pathological proliferation or harnessed for stem cell maintenance.

Taken together, these findings establish Piezo channels as hubs rather than isolated sensors: their output is gated by partner modules that decide whether mechanical cues promote vascular relaxation, immune activation, epithelial dysfunction, or tumor adaptation. Targeting Piezo–partner complexes, rather than Piezo alone, may therefore offer more precise strategies for digestive system disorders where mechanotransduction is disrupted.

### Downstream effectors and biological outcomes

The activation of Piezo channels elicits a broad range of downstream signaling cascades that govern key physiological and pathological processes. One well-characterized outcome is the stimulation of nitric oxide release, particularly through Piezo1-mediated calcium influx in endothelial cells, which activates eNOS, contributing to vasodilation and neurovascular coupling.^9^ In addition, Piezo1 regulates the activity of Yes-associated protein (YAP), a key mechanotransduction effector involved in the Hippo pathway, thereby influencing cell proliferation, tissue regeneration, and tumorigenesis;^62^ notably, Piezo1-YAP signaling has been implicated in the progression of oral squamous cell carcinoma.^63^

These insights into Piezo ion channel signaling pathways emphasize their central role in mechanotransduction and cellular homeostasis, highlighting their therapeutic potential in disorders associated with altered mechanosensitivity and signaling dysregulation.

## Expression and distribution of Piezo channels in the digestive system

The distribution of Piezo channels in the digestive system shows cell type–specific patterns that already hint at their functional specialization (Table 1). Piezo1 is widely expressed across secretory glands, epithelial compartments, hepatobiliary and pancreatic tissues, and the enteric nervous system. Its detection in gastric G cells, pancreatic acinar cells, cholangiocytes, and hepatocytes suggests its involvement in gastric secretion, enzyme release, and bile transport.^17^^,^^64^, ^65^, ^66^

**Table 1:** Expression patterns of Piezo ion channels across mammalian tissues and cell types.

Organ/Tissue	Location	Cell Type	Piezo Type	Reference
Stomach	Gastric antrum	G cells	Piezo1	64
Pancreas	Surface of pancreatic acinar cells	Acinar cells	Piezo1	17
Liver	Liver	Hepatocytes	Piezo1	65
Bile duct	Bile ducts	Ductal epithelial cells	Piezo1	66
Intestine	Small intestine and colon	Enteric epithelial cells, goblet cells, fibroblastic reticular cells (Peyer’s patches)	Piezo1	67,68,77
	Colon, small intestine	Enterochromaffin cells	Piezo1, Piezo2	15
	Small intestine, cecum, colon	Cholinergic enteric neurons (ChAT^+^)	Piezo1	74
	Small intestine (SIP syncytium)	Smooth muscle cells, interstitial cells of Cajal, and PDGFRα^+^ cells	Piezo1	13
	DRG	Sensory neurons (mechanosensory subtypes, incl. Aδ HTMRs)	Piezo2	76
	Enteric nervous system	Intrinsic primary afferent neurons (IPANs)	Piezo2	74

Within the intestinal mucosa, Piezo1 is enriched in absorptive and goblet cells, with expression higher in the colon than in the small intestine, consistent with a role in mucosal defense.^67^^,^^68^ In enterochromaffin (EC) cells, both Piezo1 and Piezo2 show method-dependent discrepancies: several single-cell transcriptomic datasets fail to consistently detect one or both isoforms in mice,^69^ whereas immunostaining, RT-qPCR and functional assays of mechanically evoked 5-HT release support their presence in both mice and humans.^70^^,^^71^ These inconsistencies likely reflect the limited sensitivity of transcriptomic approaches or isoform restriction to small subpopulations, underscoring the need for integrated single-cell spatial and functional studies.^72^ Beyond EC cells, Piezo1 senses the mechanical load in intestinal L cells to regulate GLP-1 secretion, thereby linking gut mechanotransduction to systemic metabolic control.^73^

In the muscular compartment, Piezo1 is expressed in SIP syncytia (smooth muscle cells, interstitial cells of Cajal, and PDGFRα^+^ cells), consistent with its role in Ca^2+^ oscillations and contractile rhythm.^13^ At the neuronal level, Piezo1 is broadly detected in cholinergic neurons (ChAT^+^),^74^ while Piezo2 is largely absent from enteric somata but is robustly expressed in intrinsic primary afferent neurons (IPANS) and colon-innervating dorsal root ganglion (DRG) afferents, where it senses distension and contributes to nociception and reflex control.^75^^,^^76^

Overall, Piezo1 emerges as the dominant isoform spanning glandular, epithelial, muscular, and neural compartments, whereas Piezo2 contributes to specialized sensory input via extrinsic afferents. This division of labor provides a framework for linking mechanosensory distribution to gastrointestinal motility and secretion, while also highlighting unresolved methodological and species-dependent discrepancies.

## Physiological roles of Piezo channels in the digestive system

The digestive system heavily relies on mechanical stimuli to regulate essential processes such as digestion, nutrient absorption, waste elimination, and bile secretion.^78^ Piezo ion channels, including Piezo1 and Piezo2, serve as critical mechanosensors that detect and respond to these mechanical cues, playing pivotal roles in digestive physiology. Table 2 summarizes the functional roles and underlying mechanisms of Piezo1 and Piezo2 in various cell types and tissues.

**Table 2:** Functional roles and mechanistic insights of Piezo1 and Piezo2 in specific cell types and tissues.

Piezo Type	Location/Cell Type	Physiological Function	Mechanism	Reference
Piezo	Pharynx in *C. elegans* (pezo-1 mutant)	Regulates feeding behavior	Pezo-1 mutation alters mechanosensitivity and pharyngeal pumping	81
Piezo1	Gastric G cells	Suppresses ghrelin secretion, regulates appetite	Activated by mechanical stimuli or Yoda1, reducing ghrelin secretion	79
	Visceral mechanosensory neurons (brain-gut axis)	Promotes satiety, inhibits food intake	Responds to gastric distension signals, involved in satiety signaling	80
	Smooth muscle cells, interstitial cells of Cajal, and PDGFRα^+^ cells	Maintains intestinal motility	Regulates rhythmic Ca^2+^ oscillations and contractile activity; loss of Piezo1 disrupts peristalsis	13
	Cholinergic enteric neurons (ChAT^+^) in small intestine, cecum, colon	Enhances colonic transit, supports mucosal immune homeostasis	Senses luminal pressure, promotes ACh release, enhances neuromuscular excitability	74
	Bile canalicular membranes (liver)	Facilitates bile secretion and flow	Detects bile accumulation, triggers calcium-mediated contractile responses	66
	Gut microbiota-associated tissues	Maintains microbiota composition and homeostasis	Piezo1 deficiency alters microbial community diversity and structure	86
Piezo2	Enteric enterochromaffin cells	Maintains intestinal motility and digestion	Mechanical activation induces serotonin release to regulate gut transit	82,83
	Sensory neurons innervating the gut	Regulates gastrointestinal motility	Piezo2 deficiency alters gastrointestinal transit patterns	84
	Gut microbiota-associated tissues	Mediates microbiota-regulated visceral hypersensitivity and dysmotility	Fusobacterium abundance and metabolites upregulate Piezo2, contributing to altered mechanosensitivity	88

Piezo1 contributes to appetite regulation at multiple levels. In the stomach, its activation by the agonist Yoda1 or by mechanical stimulation suppresses ghrelin secretion, reducing appetite and promoting weight loss in animal models.^79^ At the neural level, Piezo1 participates in satiety signaling through visceral mechanosensory neurons in the brain, responding to stomach distension signals and thereby curbing food intake.^80^ Evolutionary conservation of this role is evident in *C. elegans*, where mutations of the Piezo homolog (pezo-1) disrupt pharyngeal pumping and mechanosensitivity, underscoring the fundamental role of Piezo channels in feeding behaviors.^81^

Piezo channels also play a central role in gastrointestinal motility, digestion, and absorption. In EC cells, Piezo2 senses luminal mechanical stimuli and triggers calcium influx, which drives serotonin release and thereby modulates intestinal transit.^82^^,^^83^ Beyond epithelial signaling, Piezo2-mediated mechanotransduction in sensory neurons directly affects gastrointestinal motility, with Piezo2-deficient mice exhibiting altered gastrointestinal transit patterns.^84^ Complementing these roles, Piezo1 operates at the effector level. In goblet cells, it responds to shear/hydrostatic forces to activate a Ca^2+^/ERK program that up-regulates MUC2 and sustains the mucus barrier^85^; Piezo1 in SIP syncytia maintains rhythmic Ca^2+^ oscillations and contractile activity, with Piezo1 depletion leading to impaired peristalsis. In cholinergic enteric neurons, Piezo1 enables luminal pressure sensing and promotes acetylcholine release, thereby supporting neuromuscular excitability, peristaltic coordination, and mucosal immune balance.^13^^,^^74^ Extending beyond the gut, Piezo1 also localizes to bile canalicular membranes in hepatocytes, where its activation drives Ca^2+^-dependent contractions that propel bile flow.^66^ Piezo1 maintains gut dynamics through muscle, neuronal, and epithelial-immune regulation, while Piezo2 provides upstream sensory and secretory input. How these pathways converge remains a key question for motility disorders such as constipation and irritable bowel syndrome.

Additionally, Piezo1 modulates the composition and homeostasis of the gut microbiome. Piezo1 deficiency has been associated with alterations in microbial community structure, as evidenced by increased microbial diversity within mucosal-associated communities and reduced diversity in fecal-associated microbiomes.^86^^,^^87^ Beyond this, Piezo2 is also subject to microbiome regulation: recent evidence shows that Fusobacterium induces intestinal dysmotility and visceral hypersensitivity by up-regulating colonic and DRG Piezo2. These effects are associated with increased levels of indole-3-acetic acid and indole-3-propionic acid, which are predicted to interact with Piezo2, and can be alleviated by Piezo2 knockdown.^88^ Together, these findings indicate that Piezo1 shapes microbial ecosystem stability, whereas Piezo2 mediates microbe-driven mechanosensory signaling in the gut, underscoring a bidirectional Piezo–microbiota axis relevant to functional gastrointestinal disorders.

Piezo channels integrate appetite regulation, secretion, motility, and microbiota interactions into the core of digestive physiology. Piezo1 has emerged as a broad homeostatic regulator, whereas Piezo2 provides specialized sensory input. Future work should clarify how their pathways intersect across epithelial, muscular, neuronal, and microbial levels to explain digestive system diseases.

## Pathophysiological roles of Piezo channels in the digestive system

### Digestive system cancers

**Figure 3:**
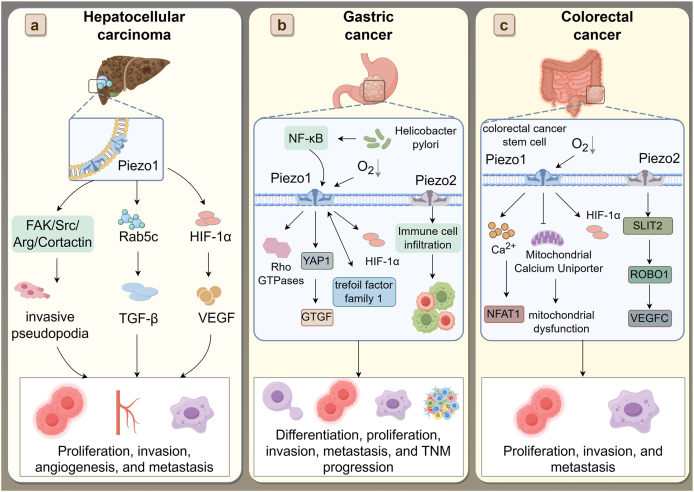
Pathophysiological Roles and Mechanistic Pathways of Piezo Ion Channels in Digestive System Cancers. **(a)** Hepatocellular carcinoma (HCC): Piezo1 expression is up-regulated in HCC and promotes tumor progression by integrating mechanical cues with signaling pathways related to invasion, angiogenesis, and metastasis. Mechanistically, Piezo1 activates the FAK/Src/Arg/cortactin axis to induce invasive pseudopodia, and enhances Rab5c-mediated TGF-β signaling as well as HIF-1α-induced VEGF expression. **(b)** Gastric cancer: *Helicobacter pylori* infection and hypoxic stress up-regulate Piezo expression, which subsequently activates the NF-κB–YAP1–CTGF axis, Rho GTPases, and HIF-1α signaling. These events contribute to increased immune cell infiltration, fibrosis, and cellular invasiveness. Trefoil factor family 1 (TFF1) and other downstream molecules further modulate tumor progression. **(c)** Colorectal cancer: Piezo1 is predominantly expressed in colorectal cancer stem cells (CCSCs) and enhances metastatic behavior via the Ca^2+^/NFAT1 pathway and mitochondrial calcium uniporter (MCU)-dependent mitochondrial dysfunction. Piezo2 promotes proliferation and migration via the SLIT2-ROBO1-VEGFC axis. Both channels support cell survival under hypoxia and contribute to tumor aggressiveness and therapeutic resistance. These findings collectively underscore the central role of Piezo channels in tumorigenesis, metastasis, immune modulation, and stromal interaction across digestive system cancers, highlighting their potential as diagnostic markers and therapeutic targets.

Digestive system cancers such as hepatocellular carcinoma, gastric cancer, and colorectal cancer remain difficult to treat, with high recurrence and resistance to conventional therapies.^89^^,^^90^ Across these malignancies, Piezo channels serve as upstream mechanotransducers that integrate peristaltic stress, luminal pressure, and matrix stiffness into conserved oncogenic signals.^91^ Ca^2+^ influx through Piezo channels activates cytoskeletal remodeling (FAK/Src–RhoA/ROCK) and transcriptional drivers (YAP/TAZ), collectively promoting invasion, proliferation, and epithelial–mesenchymal transition.^92^ Beyond tumor cells, Piezo activity also reshapes the microenvironment: in cancer-associated fibroblasts, it reinforces ECM remodeling and matrix stiffening, while in immune cells, it influences macrophage polarization and immune evasion.^93^ These shared mechanisms provide a unifying framework for understanding how Piezo channels contribute to digestive cancers, and subsequent sections detail isoform-specific and disease-specific features (Fig. 3).

#### *Hepatocellular carcinoma*

Hepatocellular carcinoma (HCC) is the sixth most common cancer globally and ranks as the fourth leading cause of cancer-related mortality.^94^ Recent studies have demonstrated that the mechanosensitive ion channel Piezo1 plays a crucial role in the progression of HCC, particularly through its involvement in angiogenesis, matrix sensing, EMT, and cell invasion.

Piezo1 expression is significantly up-regulated in HCC tissues and cell lines, and its high expression is correlated with aggressive tumor phenotypes and poor clinical outcomes.^65^^,^^95^ Functional studies have shown that activation of Piezo1 promotes proliferation, migration, invasion, and EMT in HCC cells, whereas knockdown of Piezo1 markedly suppresses tumor growth and metastasis both *in vitro* and *in vivo*.^96^^,^^97^

Mechanistically, Piezo1 functions as a key transducer of matrix stiffness. It activates the FAK/Src/Arg/cortactin signaling pathway to facilitate the formation of invadopodia—actin-rich protrusions that enable extracellular matrix degradation and tumor invasion. In parallel, Piezo1 engages the Ca^2+^/MLCK/MLC2 pathway, which works synergistically with the integrin β1/RhoA/ROCK1 axis to modulate cytoskeletal reorganization and enhance cell motility.^97^ These findings underscore Piezo1’s central role in integrating mechanical stimuli with pro-invasive signaling. In addition to promoting invasion, Piezo1 is also a key mediator of stiffness-induced angiogenesis. Its activation inhibits the ubiquitination of HIF-1α, thereby stabilizing this transcription factor and enhancing the expression of downstream angiogenic factors such as VEGF, CXCL16, and IGFBP2. Notably, Piezo1 expression is positively regulated by miR-625-5p, suggesting a stiffness–miRNA–Piezo1–angiogenesis regulatory axis in HCC.^65^^,^^96^ Furthermore, Piezo1 has been shown to promote HCC progression and EMT through activation of the TGF-β signaling pathway.^95^ This effect is mediated by the recruitment of Rab5c, a small GTPase that facilitates TGF-β signal transduction. Gene set enrichment analysis (GSEA) confirmed TGF-β signaling as a key downstream target of Piezo1 in HCC.

Nevertheless, important uncertainties remain. Most evidence still derives from hepatoma cell lines or murine xenografts, raising concerns about human translatability. A key unresolved issue is whether Piezo1 acts as an early driver of hepatocarcinogenesis under chronic mechanical stress (e.g., cirrhotic stiffness, portal hypertension) or as an amplifier of later-stage progression through angiogenesis and EMT. In addition, the near absence of data on Piezo2 further complicates the understanding of isoform-specific roles or potential compensation. Taken together, Piezo1 is increasingly positioned as a central mechanical hub in HCC, but defining its causal role and therapeutic tractability will require physiologically relevant models that capture the fibrotic–inflammatory liver microenvironment.

#### *Gastric cancer*

Gastric cancer is characterized by late diagnosis and low survival rates, highlighting an urgent need for novel therapeutic targets and biomarkers for early detection and treatment.^98^ Recent studies have underscored the significant role of Piezo ion channels, especially Piezo1 and Piezo2, in the pathophysiology of this disease.

Piezo2 is emerging as a clinically relevant mechanosensor in gastric cancer. Its expression is markedly elevated in tumor tissues and is correlated with advanced TNM stage, lymph node metastasis, and poor survival, positioning it as an independent prognostic biomarker.^99^ Beyond prognosis, transcriptomic analyses link Piezo2 expression in gastric cancer to immune infiltration signatures, especially elevated macrophages (M2) and certain T-cell subsets, suggesting that Piezo2 may shape the tumor immune microenvironment. However, these findings are based on bioinformatic correlation, and experimental validation is needed to confirm causality.^100^ Although direct mechanistic studies remain scarce, these associations imply that Piezo2 may act at the interface between mechanical cues and immune modulation, a dimension less explored for Piezo1. Thus, Piezo2 not only reflects tumor aggressiveness but also emerges as a potential immune-oriented therapeutic target in gastric cancer.

Transitioning to Piezo1 influences gastric cancer progression through several pathways. It is highly expressed in gastric cancer cells and contributes to the promotion of cell proliferation and migration. Piezo1 modulates the activity of Rho GTPases, enhancing their cellular invasive capabilities.^101^ This mechanosensitive ion channel also responds to cues from the microenvironment, such as those provided by the presence of *Helicobacter pylori*, which activates NF-κB signaling, further up-regulating Piezo1 expression and promoting cancer progression. The NF-κB-Piezo1-YAP1-CTGF axis, stimulated by *H. pylori*, is critical for remodeling the tumor microenvironment by facilitating the infiltration of cancer-associated fibroblasts and enhancing fibrosis around tumor cells, thus supporting cancer cell survival and metastasis.^102^ Targeting this pathway has shown promise, as evidenced by the enhanced efficacy of chemotherapeutic agents like 5-fluorouracil when Piezo1 is knocked down or its pathway is pharmacologically inhibited.^101^

Furthermore, the role of Piezo1 extends to adapting to hypoxic conditions within tumors. It regulates the expression of HIF-1α. By modulating the cellular response to hypoxia, Piezo1 not only supports cancer cell survival under low oxygen conditions but also promotes their motility and invasiveness, which are essential for metastatic spread.^18^ Additionally, the interaction between Piezo1 and trefoil factor family 1 (TFF1) underscores its role in enhancing gastric cancer cell mobility and aggressiveness, further supporting its potential as a therapeutic target to curb gastric cancer progression and metastasis.^103^

These insights into the functions of Piezo channels in gastric cancer suggest that they actively drive disease progression by promoting tumor cell proliferation, invasion, hypoxia adaptation, and tumor-associated fibrosis. Their capacity to integrate mechanical cues with inflammatory and stromal signaling highlights their dual potential as biomarkers of aggressive phenotypes and as promising therapeutic targets in gastric cancer. Future studies should specifically address how H. pylori-induced inflammatory signaling intersects with the Piezo1-YAP/CTGF pathways, and whether Piezo2-linked immune infiltration patterns can be harnessed to stratify patients for immunotherapy. These questions are central to translating Piezo biology into clinically meaningful strategies in gastric cancer.

#### *Colorectal cancer*

Colorectal cancer, characterized by its asymptomatic nature in the early stages, often leads to diagnoses in advanced stages where patient outcomes are notably poor.^104^ Piezo2 is significantly up-regulated in colorectal tissues and is correlated with diminished overall survival, suggesting its crucial role in the progression of this malignancy.^105^

Piezo1 is predominantly expressed in colorectal cancer stem cells (CD133^+^/CD44^+^), where it enhances self-renewal and colony formation, supporting initiation, recurrence, and metastatic competence.^106^ Functionally, Piezo1 promotes migration and invasion through Ca^2+^/NFAT1 signaling.^106^ Notably, primary (SW480) and metastatic (SW620) cells exhibit differential Piezo1-dependent Ca^2+^ responses and apoptotic sensitivity under fluid shear stress, underscoring the heterogeneity of Piezo1 signaling in tumor progression.^107^ By contrast, Piezo2 supports cellular proliferation, migration, and invasion through the SLIT2/ROBO1/VEGFC pathway, reinforcing lymphangiogenesis and metastatic dissemination.^105^

The complex interplay between Piezo1 and hypoxia-related pathways also merits attention. The overexpression of Piezo1 can override the suppression of migration typically induced by HIF-1α inhibition.^108^ This dynamic is reflected in the mitochondrial dysfunction commonly observed in colorectal cancer, where Piezo1 appears to target the mitochondrial calcium uniporter (MCU) for down-regulation. Conversely, silencing MCU enhances the vitality and metastatic capability of cancer cells, presenting a nuanced relationship that affects tumor behavior and progression.^108^

Taken together, the current evidence highlights Piezo1 as a central driver of colorectal cancer stemness, invasion, and hypoxia-mitochondrial adaptation, while Piezo2 promotes lymphangiogenesis and nodal dissemination through SLIT2/ROBO1/VEGFC signaling. Unlike HCC or gastric cancer, CRC thus features a dual reliance on CSC maintenance and lymphatic spread, processes in which Piezo channels appear particularly critical. Future work should determine whether targeting Piezo1 in CCSCs or disrupting Piezo2-driven lymphangiogenesis can effectively mitigate recurrence and metastatic progression in colorectal cancer.

## Pancreatitis

Elevated pancreatic pressure is a primary cause of acute pancreatitis, often resulting from abdominal trauma, surgical interventions, endoscopic retrograde cholangiopancreatography (ERCP), and complications from gallstones.^109^^,^^110^ The regulation of intracellular calcium levels is crucial for maintaining the homeostasis of acinar cells, which are specialized cells in the pancreas that release digestive enzymes.^111^ Dysregulation of calcium levels can lead to mitochondrial dysfunction, premature activation of digestive enzymes within cells, and ultimately cell necrosis, all of which contribute to the development of pancreatitis.^112^

Piezo1 has emerged as a pivotal mechanosensor in this process. Mechanical stress during procedures or pressure elevation opens Piezo1 channels, permitting sustained Ca^2+^ entry that drives both mitochondrial dysfunction and early conversion of trypsinogen to trypsin.^17^^,^^113^^,^^114^ Strategies to block Piezo1 have shown promising results in reducing pancreatitis severity. Pharmacological inhibition with GsMTx-4 or genetic deletion of Piezo1 in pancreatic acinar cells markedly reduces pancreatic damage in mouse models, highlighting Piezo1 as a trigger of pressure-induced pancreatitis and a candidate therapeutic target.^17^

Importantly, Piezo1 does not act alone. Pressure-evoked Piezo1 activity engages TRPV4, another calcium-permeable channel, amplifying calcium overload and downstream injury cascades.^54^ TRPV4 knockout mice are resistant to Piezo1-driven pathology, establishing Piezo1–TRPV4 coupling as a synergistic module that converts mechanical stress into irreversible acinar damage. This interaction exemplifies how mechanotransduction can function as a feed-forward amplifier of organ injury rather than a linear pathway.^54^

Despite compelling evidence from rodent models, whether Piezo1 also contributes to basal acinar secretion or is compensated by Piezo2 and other channels remains unclear, raising caution for its therapeutic targeting. Conceptually, Piezo1 emerges as a mechanosensitive trigger of calcium overload and acinar injury, often amplified through TRPV4, yet its clinical translation will require validation in human tissues and careful assessment of prophylactic blockade in high-risk settings such as ERCP.

## Inflammatory bowel disease

Inflammatory bowel disease (IBD) includes Crohn’s disease and ulcerative colitis, characterized by persistent intestinal inflammation.^115^ Mechanosensitive Piezo channels have emerged as key mediators linking mechanical stress to epithelial, immune, and neuronal responses in the inflamed gut. While Piezo1 primarily regulates inflammation and barrier integrity, Piezo2 contributes to visceral mechanosensation and pain, together shaping IBD pathophysiology.^116^^,^^117^

Research has found that Piezo1 is highly expressed in the ileum of Crohn’s disease patients and is significantly correlated with disease activity indicators such as the CDAI score and fecal calprotectin ^118^. Mechanistically, Piezo1 activation triggers ROS accumulation and mitochondrial dysfunction, thereby activating the NLRP3 inflammasome and inducing pro-inflammatory cytokines; these effects can be blocked by GsMTx4 or Piezo1 siRNA.^118^ Piezo1 also activates NF-κB through the Ca^2+^/CaMKII dependent pathway, aggravating inflammation in models such as necrotizing enterocolitis.^16^ More recently, Piezo1 was shown to exacerbate colitis by engaging the Nrf2/NF-κB/NLRP3 axis, thereby linking mechanical stress to oxidative stress and inflammasome activation.^119^

In addition to the role in inflammation amplification, Piezo1 also participates in the regulation of intestinal barrier function. Its sustained activation can induce lipid peroxidation and ferroptosis by inhibiting the AMPK/mTOR pathway, leading to the loss of tight junction proteins and increased barrier permeability.^120^ Piezo1 also exacerbates the course of IBD by affecting mucosal immunity and microbiota homeostasis. In macrophages, Piezo1 activation promotes M1 polarization and activation of the NLRP3/NF-κB pathway, increasing the secretion of inflammatory factors.^121^ In addition, Piezo1 regulates the expression of IL-17A in ILC3 through the PI3K/Akt/mTOR pathway, participating in innate immune regulation.^122^ At the microbiota level, Piezo1-deficient mice showed a decrease in FAM diversity and an increase in potential pathogenic bacteria such as Shigella in MAM, indicating their impact on the gut microbiota through barrier stability.^86^ Once the barrier is disrupted and the microbiota is disrupted, a positive feedback loop continues to drive the development of inflammation.

Beyond epithelial and immune mechanisms, recent studies have highlighted the contribution of Piezo2 in sensory neurons to IBD pathophysiology. Colon-innervating DRG neurons comprise genetically defined subtypes with distinct mechanosensory thresholds, among which Aδ high-threshold mechanoreceptors (HTMRs) are critically involved in nociceptive responses to colonic distension.^76^ This population relies partly on Piezo2 activity and exhibits exaggerated sensitivity under inflammatory conditions, leading to behavioral hyper-responsiveness to colonic distension in IBD models.

In summary, Piezo1 promotes IBD progression through Nrf2/NF-κB/NLRP3 activation, ROS accumulation, and barrier disruption, whereas Piezo2 enhances mechanosensory signaling that drives visceral hypersensitivity. What remains unresolved is whether these mechanistic pathways initiate chronic mucosal remodeling or primarily reinforce established inflammation, a distinction that will be critical for translating Piezo targeting into IBD-specific therapeutic strategies.

## Functional gastrointestinal disorders

Functional gastrointestinal disorders (FGIDs), including irritable bowel syndrome (IBS), functional dyspepsia, and functional constipation, affect nearly 40% of the global population.^123^ Despite their high prevalence, the mechanisms underlying visceral hypersensitivity, motility disturbances, and barrier dysfunction remain incompletely understood. Emerging evidence implicates mechanosensitive Piezo channels as pivotal transducers linking mechanical stimuli to these pathophysiological processes.^124^

Piezo2 is increasingly recognized as a key mediator of colonic mechanosensation and visceral hypersensitivity. In EC cells, Piezo2 activation drives Ca^2+^ influx and serotonin release, thereby amplifying hypersensitivity in IBS models; conversely, Piezo2 knockout alleviates these symptoms, supporting its potential as a therapeutic target.^15^ In sensory neurons, Piezo2 functionally couples with TRPV1 channels to enhance nociceptive signaling under pathological conditions.^56^ External modulators further shape its activity: laminarin attenuates hypersensitivity in a dyspepsia model by normalizing Piezo2–EPAC1 signaling and 5-HT_3_ receptor expression,^125^ while satellite glial cells in the DRG up-regulate neuronal Piezo2 to sustain mechanical hypersensitivity.^126^ Recent work has highlighted sex- and age-dependent differences in Piezo2-driven sensitivity, though the underlying mechanisms remain unresolved.^127^ These findings establish Piezo2 as both an epithelial and a neuronal mechanosensor whose activity is tuned by metabolic, glial, and demographic factors, positioning it as a context-dependent driver of FGIDs.

Piezo1 contributes to intestinal barrier regulation by modulating tight junction proteins and mucosal permeability. Under mechanical stress, such as fecal flow, its overactivation increases permeability, a key feature of IBS, through ROCK-dependent down-regulation of claudin-1 and related junctional components, thereby compromising epithelial defense.^128^

Both Piezo1 and Piezo2 contribute to motility regulation. Microbe-derived single-stranded RNA (ssRNA) acts as a natural Piezo1 ligand, stimulating serotonin synthesis in a MyD88/TRIF-independent manner and affecting gut motility.^36^ This ssRNA-Piezo1 interaction emphasizes the role of microbial by-products in gut physiology and disease. In parallel, Piezo2-mediated mechanotransduction in EC cells enhances serotonin release in response to luminal distension, further influencing motility patterns.^70^ Our recent study experimentally demonstrated that the knockdown of Piezo1 or Piezo2 in EC cells reduces 5-HT release and exacerbates dysmotility in functional constipation models, while simultaneous knockdown produces an additive effect, underscoring their cooperative role in gut motility regulation.^71^ Together, these findings highlight how Piezo channels integrate microbial and mechanical cues into a coordinated serotonergic network that governs intestinal transit.

Taken together, Piezo2 has emerged as a key driver of visceral hypersensitivity, whereas Piezo1 shapes barrier function and microbial signaling. Both converge on serotonin pathways to fine-tune gut motility, positioning Piezo channels as integrators of sensory, epithelial, and microbial cues.

## Clinical translation prospects of targeting Piezo channels

Clinical exploration of Piezo channels in digestive diseases is still nascent, with current evidence largely confined to retrospective tissue cohorts and preclinical models; prospective validation in well-characterized patient populations is still lacking.^129^ In digestive cancers, high Piezo1 expression is correlated with poor prognosis in hepatocellular carcinoma and colorectal cancer,^65^^,^^95^ whereas Piezo2 levels are linked to immune infiltration signatures in gastric cancer.^99^^,^^105^ In Crohn’s disease, Piezo1 is up-regulated in the ileum and correlates with the CDAI and fecal calprotectin, suggesting its potential as a biomarker, though longitudinal validation is lacking.^118^ These associations highlight translational promise but underscore the need for prospective studies in well-characterized patient populations.

Current pharmacological tools provide preclinical proof of concept. The Piezo1 agonist Yoda1 enhances epithelial defense, while inhibitors such as GsMTx4 and Dooku-1 attenuate calcium overload and tissue injury in colitis and pancreatitis models.^130^^,^^131^ However, the broad physiological distribution of Piezo1 across vascular, hematopoietic, skeletal, and renal systems means that systemic modulation risks off-target toxicity, including hemolysis, barrier dysfunction, or bone abnormalities.^132^, ^133^, ^134^ These challenges emphasize the urgent need for isoform-selective modulators coupled with targeted delivery systems, such as gut-restricted formulations, liver-directed nanoparticles, or receptor-guided conjugates, to achieve therapeutic precision and safety.^135^

Beyond pharmacology, gene- and RNA-based interventions are being explored as long-term strategies. Conditional knockout studies have revealed that smooth muscle–specific Piezo1 deletion impairs the peristaltic rhythm,^13^ enteroendocrine-specific loss alters GLP-1 secretion,^73^ and DRG-specific Piezo2 manipulation modifies visceral nociception, demonstrating the feasibility of cell-type–restricted targeting.^76^ Inducible RNA interference, CRISPRi, and lipid nanoparticle–mediated siRNA delivery provide routes for transient, organ-specific modulation.^136^^,^^137^ In parallel, advanced tools such as HaloTag-based imaging in hiPSC-derived organoids enable real-time visualization of Piezo activity, bridging mechanistic discovery with translational testing.^138^ Taken together, these lines of evidence underscore both the therapeutic promise and translational challenges of Piezo channel targeting, highlighting precision, selectivity, and human-relevant validation as key priorities for clinical application.

## Conclusion

Recent investigations into Piezo ion channels have profoundly enhanced our understanding of mechanotransduction in the digestive system. These channels regulate critical functions, such as appetite, digestion, absorption, bile flow, and intestinal microbial balance, and are implicated in various gastrointestinal disorders, including digestive cancers, inflammatory diseases, and functional bowel disorders. While this review highlights the significant roles and therapeutic potential of Piezo channels, specific gaps must be addressed to fully exploit their capabilities in clinical settings.

1. Human physiological and pathological contexts: The current understanding of Piezo channels in the digestive system is built largely on rodent models and simplified *in vitro* assays, which cannot fully recapitulate the immunological, microbial, and structural complexity of human tissues. This limitation is especially critical in IBD and digestive cancers, where the microenvironmental context decisively shapes disease trajectories and treatment responses. Advanced patient-derived platforms, such as tumor organoid biobanks and gut organoid–immune co-culture systems, now provide more physiologically relevant contexts to study Piezo function and may help bridge the translational gap.
2. Targeted therapeutic development: The therapeutic promise of Piezo modulation is increasingly evident, yet its clinical translation is constrained by its broad tissue distribution and the absence of isoform-specific modulators. Non-selective pharmacological interventions risk unintended effects on vascular, hematopoietic, or skeletal systems. These concerns highlight precision and safety as central priorities. Future progress depends on isoform-selective modulators combined with delivery strategies that restrict activity to defined tissues or cell types. Advances in structural pharmacology, molecular imaging (e.g., HaloTag real-time tracking of Piezo activity), and gene- or RNA-based approaches (e.g., CRISPRi, LNP-mediated siRNA delivery) provide tools for more selective and temporally controlled modulation, but rigorous preclinical validation remains essential.
3. Complex molecular interaction networks: Piezo channels operate not as isolated sensors but as nodes in complex molecular networks, coupling Ca^2+^ influx to cytoskeletal remodeling, YAP/TAZ transcriptional programs, and immune–stromal interactions. These interconnected pathways are context dependent: protective in barrier maintenance and epithelial repair, but pathogenic when driving tumor invasion, fibrosis, or chronic hypersensitivity. Future research should prioritize high-resolution mapping of Piezo-centered interactomes in patient-derived systems to clarify whether Piezo activity represents a driver of pathology or an adaptive response.

In conclusion, while the role of Piezo ion channels in the digestive system is increasingly recognized, addressing these research deficiencies is crucial for developing novel therapeutic strategies that leverage the unique properties of these mechanosensitive channels. By filling these gaps, future research can transform our approach to managing digestive diseases, improving patient outcomes through targeted and effective treatments.

## CRediT authorship contribution statement

**Xiangyun Yan:** Writing – review & editing, Writing – original draft, Formal analysis, Data curation. **Weijian Zeng:** Writing – review & editing. **Peitao Ma:** Visualization. **Junpeng Yao:** Writing – review & editing. **Tingting Ma:** Investigation. **Ying Li:** Funding acquisition, Conceptualization.

## Funding

This review was supported by the 10.13039/501100001809National Natural Science Foundation of China (No. 82274652).

## Conflict of interests

The authors declare that they have no competing interests for this article.
